# Drug resistant epilepsy and associated factors among children with epilepsies in Tanzania: a cross-sectional study

**DOI:** 10.1186/s12883-023-03508-9

**Published:** 2024-01-02

**Authors:** Obrey H Urio, Edward Kija, Sarah Weckhuysen, Hilda Makungu, Helga Naburi

**Affiliations:** 1 Department of Paediatrics and Child Health, Muhimbili University of Health and Allied Sciences, Dar es Salaam, Tanzania; 2 University of Antwerp, Antwerp, Belgium; 3 Department of radiology, Muhimbili National Hospital, Dar es Salaam, Tanzania

**Keywords:** Epilepsy, Drug resistant, Seizures, Anti-seizure medications, International league against epilepsy

## Abstract

**Background:**

Epilepsy contributes to high morbidity among children and adolescents in developing countries. A quarter of all children with epilepsy will be resistant to anti-seizure medications (ASMs), with associated neurocognitive impairments and risk of higher mortality. This study aimed to estimate and characterize drug-resistant epilepsy (DRE) (defined as failure to achieve sustained remission after adequate trials of two tolerated and appropriately chosen ASMs) and its associated factors among children and adolescents with epilepsies attending the pediatric neurology clinic at Muhimbili National Hospital (MNH), Dar es Salaam Tanzania.

**Methods:**

This cross-sectional study was conducted from June 2020 to June 2021. Children with epilepsies and who had been treated with ASMs for at least 3 months were eligible for inclusion. Exclusion criteria included children whose caregivers denied consent and those who exhibited acute medical conditions necessitating admission on the scheduled visit day. Data on demographic characteristics, perinatal history, detailed history of the seizures semiology, drug history, magnetic resonance imaging (MRI), and electroencephalography (EEG) results were obtained from caregivers and medical records available during recruitment. Seizures and epilepsies were classified using the 2017 International League Against Epilepsy (ILAE) classification. Logistic regression was used to determine factors associated with DRE.

**Results:**

A total of 236 children and adolescents aged between 4 months and 15 years (Median age 72 months (IQR = 42–78)) were enrolled in this study. We found the proportion of DRE to be 14.8% in this cohort. Of the thirty-five patients with DRE, 60% had generalized epilepsy and almost 25% had a diagnosis of an epilepsy syndrome, the most common being Lennox-Gastaut syndrome (LGS). Structural abnormalities on brain MRI were seen in almost 80% of all patients with DRE, the most prevalent being cystic encephalomalacia, which was observed in 34% of patients. Patients using both ASMs and alternative therapies accounted for 9% of this cohort. The onset of seizures during the first month of life (aOR = 1.99; 95%CI 1.7–4.6; p = 0.031) and high initial seizure frequency (aOR = 3.6; 95%CI 1.6-8;p = 0.002) were found to be independently associated with DRE.

**Conclusion:**

The proportion of DRE in Tanzania is high. Patients with neonatal onset seizures and high initial seizure frequency should be followed up closely to ensure early diagnosis of DRE.

## Background

Epilepsy is one of the most common neurological disorders worldwide with low and middle income countries (LMIC) being disproportionally more affected [1]. Children bear a significant burden of epilepsies with more than 90% of all newly diagnosed cases being in young people aged 20 years and under [2]. Despite treatment with a variety of both old and newer anti-seizure medications (ASMs), such as sodium valproate, carbamazepine, levetiracetam and lamotrigine, between 19% and 30% of children will continue to have treatment-resistant, debilitating seizures [3–5]. Drug-resistant epilepsy (DRE) is defined as the failure to achieve seizure remission after an adequate trial of two tolerated and appropriately chosen ASMs [6]. Recurrent seizures, irrespective of the etiological cause, may lead to cognitive decline, increased risk of injuries, and ultimately reduced quality of life for the patient. Therefore, the primary goal in treating epilepsy is attaining freedom from seizures [7, 8]. Identifying children that will develop DRE at initial presentation is challenging, however, several predictors of drug resistance have been described in different settings. These include early age of onset, high seizure frequency, multiple seizure types, the etiology of epilepsy (e.g. structural abnormalities and inborn metabolism errors), developmental delay (motor and cognitive) and abnormalities detected by electroencephalogram (EEG) and neuroimaging [9–12].

Despite LMIC representing most of the worldwide epilepsy burden, there is a paucity of data on the magnitude of DRE in children in these areas. This study aimed to determine the prevalence of DRE, clinical patterns of epileptic seizures, and factors associated with DRE among children and adolescents with epilepsies attending a pediatric neurology clinic at Muhimbili National Hospital (MNH), Tanzania.

## Materials and methods

This hospital-based cross-sectional study was conducted at Muhimbili National Hospital (MNH) in Dar es Salaam, Tanzania, from June 2020 until June 2021, and was approved by the Muhimbili University of Health and Allied Sciences (MUHAS) Ethical Committee for Research and Publications (MUHAS-REC-04-2020-266). The MNH pediatric neurology clinic serves approximately one hundred and fifty patients per week, of which at least 60% have seizure disorders. Pediatric neurologists and pediatricians prescribe ASMs based on seizure type. Dosages are calculated based on body weight and patients are initially given two weekly follow-ups. ASMs are sequentially titrated based on seizure frequency as reported by the caregiver. Patients with frequent seizures are tried on another ASM within a few months after failure to achieve seizure control with maximum tolerable dose of the pervious medication.

### Inclusion and exclusion criteria

All children and adolescents aged between 4 months and 15 years diagnosed with epilepsies that were attending the pediatric neurology clinic at MNH were eligible for inclusion in the study. The number of seizures at onset was defined as episodes of seizures per day or month before ASM initiation. Patients who had at least 10 seizure episodes per day on most days before initiation of ASM were defined as having high initial seizure frequency. All cases that fulfilled the criteria for DRE were reviewed with pediatric neurologists and the Principal Investigator to make sure there was adequate information needed to reach such a conclusion. Patients whose caregivers did not consent, those with acute medical conditions necessitating admission on the day of the visit and those who were on treatment with ASMs for less than three months were excluded from the study. Seizures and epilepsies were defined as per the International League Against Epilepsy, 2017 [13].

### Data collection

Demographic and clinical data were obtained from the caregivers and patient’s electronic medical records using a standardized questionnaire. A research assistant, a medical officer working on the neurology unit, a certified pediatric epilepsy trainee (PET) registered with the British Pediatric Neurology Association (BPNA), and the Principal Investigator administered the questionnaire to the caregivers. After completion of the questionnaires on clinic days, the Principal Investigator inspected the tool for completion. Assessment of developmental milestones was conducted across all domains (i.e., gross motor, fine motor, language and communication, social emotional and cognitive) and categorized as normal, delayed, or regressed based on clinical judgment. Detailed history of medication type and dosage was obtained. Appropriateness of the ASM was assessed using a simple guide adopted from a pragmatic algorithm to select ASMs in patients with epilepsy [14]. As per standard guidelines, a period of at least three months was permitted after the maximum tolerable dose was reached before the decision of treatment failure was made. Patients were categorized as having drug-resistant epilepsy if they were not seizure-free for a duration longer than three times the pre-treatment inter-seizure interval after an adequate trial of two tolerated and appropriately chosen ASMs taken at tolerable doses [6]. Brain magnetic resonance imaging (MRI) and electroencephalogram (EEG) was not performed in all patients since these were ordered at the discretion of the primary physician. All patients with DRE had brain MRIs done and we dichotomized the findings into normal or abnormal (as determined by an experienced radiologist), followed by the radiological diagnosis. We extracted EEG reports that were performed as part of routine care. The findings were entered in the research tool as normal or abnormal EEG followed by the type of interictal epileptiform discharges.

### Sample size calculation

Sample size was calculated manually using Kish Leslie formula (1965). The prevalence used was 17.2%, which was the proportion of DRE among children with epilepsy in Nigeria [12]. With assumption of margin of error of 5% at 95% confidence level and 8% non-response rate, the minimum sample size required to power this study by 80% was 236.

### Statistical analysis

Data analysis was performed using SPSS (IBM Corp., Version 25.0, 2017). The Shapiro-Wilk test was performed to assess for normality of continuous variables such as age of the participants and age of onset of seizures. Associations between drug resistant epilepsy and clinical-demographic characteristics such as sex, age of onset of seizures, seizure types and neuroimaging findings was compared using chi-square and Fisher exact test. Univariate and multivariate logistic regression models were used to determine the factors associated with DRE. Factors with a probability value (p-value) of less than 0.2 in the univariate analysis were used to create the final multivariate model. Results were reported as odds ratios (ORs) and 95% confidence intervals (CI). A p-value < 0.05 was considered statistically significant.

## Results

### Patient characteristics

Data from a total of 236 patients (56.4% male and 43.6% female) age range 4 months – 15 years (Median age 72 months (IQR = 42–78) were included in the analysis.

### Seizure type and frequency

**Fig. 1:**
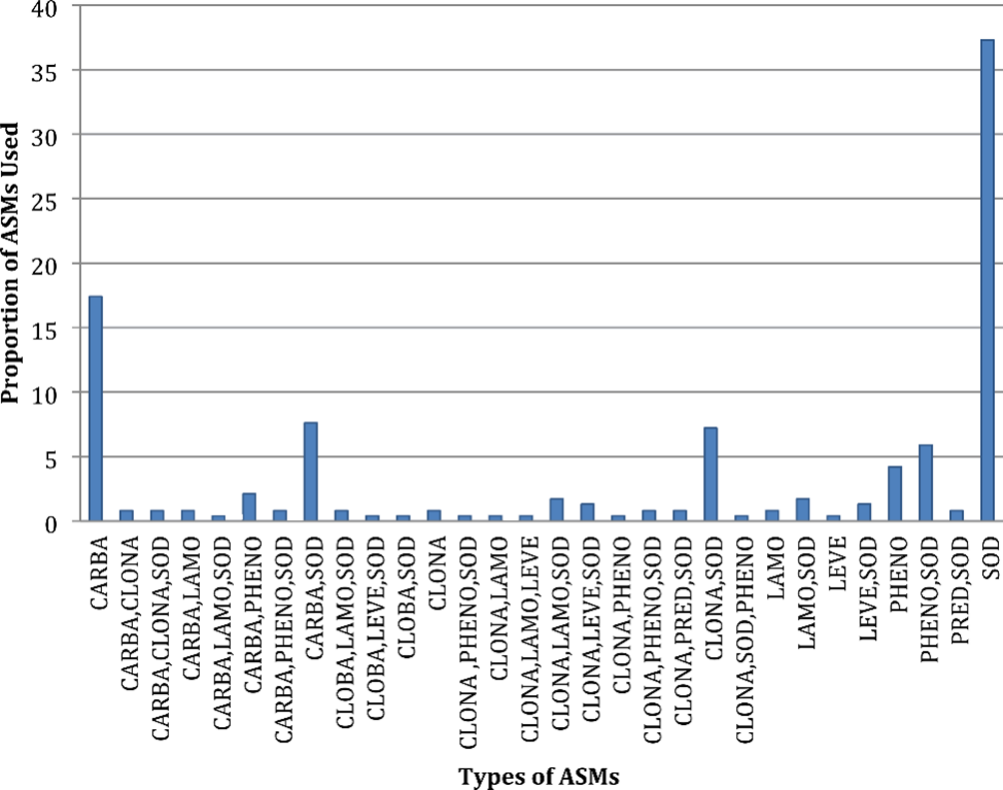
Types of Anti-seizure medications used by the study participants. Key: CLOBA-Clobazam, LEVE-levetiracetam, SOD-sodium valproate, CARBA-Carbamazepine, LAMO-lamotrigine, LEVE-levetiracetam, PHENO-phenobarbitone, PRED-prednisolone and CLONA-clonazepam

The median age of seizures onset was 7 months (IQR 1–24). Over 60% of the patients (144/236) had their first episode of seizure within the first year of life with at least a quarter of them during the neonatal period. The majority of the participants 143 (60%) had been on treatment with one ASM. The commonly used ASM as monotherapy was sodium valproate and carbamazepine followed by phenobarbitone by 88 (37.3%), 41 (17.4%) and 10 (4.2%) respectively. In patients who were on polytherapy the most frequently used combinations were carbamazepine with sodium valproate followed by sodium valproate with clonazepam (Fig. 1). Twenty-six patients (11%) were determined to not be on an appropriate ASM. This group included patients who had atonic and or myoclonic seizures and who were on carbamazepine as part of their regimen. Caregiver reported adherence was good in 223 (94.5%). Alternative therapies, such as spiritual interventions (religious and traditional rituals) and local herbs (e.g. mvuje *(Ferula assa-foetida)*) in addition to ASM were used by 22 (9.3%) of the study participants. Of the included patients, 7 (3%) were born from consanguineous parents. More than two thirds had generalized epilepsy 159 (67.4%) and 38 (16.1%) of the participants were diagnosed with infantile or childhood-onset epilepsy syndromes **(**Table 1**)**.

**Table 1:** Demographic and clinical characteristics of the study participants

Variable	Category	N	Percentage (%)
Sex	Male	133	56.4
	Female	103	43.6
Age of the participants (Years)	< 5	89	37.7
	5–10	105	44.5
	> 10	42	17.8
Age of onset of seizures (Months)	< 1	65	27.5
	1–12	79	33.5
	> 12	92	39
Number of medications currently using	One	143	60.6
	Two	73	30.9
	Three or more	20	8.5
Appropriateness of ASM	Yes	210	89
	No	26	11
Caregiver-reported adherence	Good	223	94.5
	Bad	13	5.5
Use of alternative therapies concurrently	Yes	22	9.3
	No	214	90.7
Consanguinity	Yes	7	3
Epilepsy Type	Generalized	159	67.4
	Focal	64	27.1
	Combined	13	5.5
Epilepsy Syndromes	Yes	38	16.1
	No	198	83.9

## Proportion of drug-resistant epilepsy

The proportion of DRE in this cohort was 14.8%. Patients aged between five and ten years constitued the majority of the DRE cases by 46.8% followed by those younger than five years old 37.8%. Of these, only 3 (8%) were on monotherapy, while the rest were on two to three ASMs in combination. Among those who were on polytherapy (32 (91.4%)), the most frequent drug combination was clonazepam with sodium valproate, followed by carbamazepine with sodium valproate, used by 9 (25.7%) and 5 (14.3%) patients respectively. Among patients with DRE, 21 (60%) were diagnosed with generalized epilepsies and 8 (22.9%) had a diagnosis of infantile or childhood-onset epilepsy syndrome, with Lennox Gaustat syndrome being the most common 5 (14.2%) followed by West 2 (5.7%) and Doose syndrome 1 (2.9%) **(**Table 2).

**Table 2:** Clinical patterns of seizures among patients with drug-resistant epilepsy attending neurology clinic at MNH (N = 35)

Variable	Category	N	Frequency (%)
Epilepsy type	Generalized	21	60
	Focal	11	31.4
	Combined	3	8.6
Epilepsy syndromes*	Yes	8	22.9
	No	27	77.1
Syndrome type	Doose Syndrome	1	2.9
	**Lennox-Gastaut syndrome**	5	14.2
	West syndrome	2	5.7

Epilepsy syndromes*- Diagnosis was made as per the 2017 ILAE definition of epilepsy syndromes

### EEG and MRI findings in patients with drug-resistant epilepsy

All patients with DRE had MRIs performed and more than 80% were shown to have abnormal findings. Multi cystic encephalomalacia was the most common abnormal finding (34%) followed by brain atrophy (20.6%) **(**Table 3).

The most common MRI-defined lesions among the 11 patients with focal DRE were gliosis (noted in various areas of the brain) in 4 (36.4%) followed by brain atrophy 3 (27.2) and ischemic infarcts in 2 (18.2%) of patients respectively. Two of the three patients that had combined epilepsy were found to have cystic encephalomalacia and one had focal gliosis. At least 80% of children with DRE had an abnormal interictal EEG.

**Table 3:** Neuroimaging and EEG findings among patients with drug-resistant epilepsy (N = 35)

Variable	Category	Number	Percentage (%)
EEG	Normal	6	17.1
	Abnormal	29	82.8
MRI	Normal	7	20
	Abnormal	28	80
MRI abnormalities (N = 28)	Congenital structural anomalies	2	7.1
	Gliosis	5	17.9
	Multi Cystic encephalomalacia	10	35.7
	Vascular (stroke)	2	7.1
	Hydrocephalus (Post meningitis)	1	3.6
	Brain atrophy	6	21.4
	Mesial temporal lobe sclerosis	2	7.1

## Factors associated with DRE among patients with epilepsy attending the pediatric neurology outpatient clinic at MNH

As shown in Table 4, patients were significantly more likely to develop DRE if they had neonatal onset seizures (p = 0.007), developmental delay (p = 0.017), history of status epilepticus (p < 0.001), and abnormal MRI findings (p = 0.003). Furthermore, patients with DRE were more likely to present with high seizures frequency from the onset of disease (P < 0.001).

**Table 4:** Factors associated with the development of drug-resistant epilepsy

		Drug-Resistant Epilepsy		
Variable	Category	Yes%	No%	P value
Sex	Male	20(15)	113(85)	0.9
	Female	15(14.6)	88(85.4%)	
Seizure types	1	24(12.8)	164(87.2)	0.077
	≥ 2	11(22.9)	37(77.1)	
Number of seizures at onset.	< 10	12(7.7)	144(92.3)	< 0.001
	≥ 10	23(28.7)	57(71.3)	
Developmental delay**	Yes	24(20.3)	94(79.7)	0.017
	No	11(9.3)	107(90.7)	
Neonatal seizures	Yes	16(25.8)	46(74.2)	0.007
	No	19(10.9)	155(89.1)	
Epilepsy syndrome	Yes	8(21.1)	30(78.9)	0.23
	No	27(13.6)	171(86.4)	
History of status epilepticus	Yes	20(27.4)	53(72.6)	< 0.001
	No	15(9.2)	148(90.8)	
Consanguinity	Yes	1(14.3)	6(85.7)	0.31*
	No	34(14.8)	195(85.2)	
MRI (N = 141)	Normal	7(12.1)	51(87.9)	0.003
	Abnormal	28(33.7)	55(66.3)	
EEG (N = 169)	Normal	6(13.3)	39(86.7)	0.281
	Abnormal	29(23.3)	95(76.7)	

*Fisher’s exact Test

**Children who exhibited delay in at least two of the developmental domains (i.e., gross motor, fine motor, language and communication, social emotional and cognitive)

## Independent factors associated with drug-resistant epilepsy

As shown in **(**Table 5**)**, high initial seizure frequency (aOR = 3.6; 95%CI 1.6-8; p = 0.002) and the onset of seizures during the first month of life (aOR = 1.99; 95%CI 1.7–4.6; p = 0.031) were found to be independently associated with DRE.

**Table 5:** Independent factors associated with drug-resistant epilepsy

Variable		Univariate analysis			Multivariate analysis		
	Category	COR	95% CI	P-value	AOR	95% CI	P-value
History of resuscitation	Yes	1.15	0.1–2.57	0.7			
	No						
Number of seizures at onset	> 10	4.06	2.2-10.37	< 0.001	3.6	1.6-8	0.002
	≤ 10						
Seizure onset ≤ 1 month	Yes	2.26	1.07–4.77	0.006	1.99	1.7–4.6	0.031
	No						
Developmental delay	Yes	2.3	1.15–5.34	0.02	1.8	0.8-4	0.12
	No						
History of status Epilepticus	Yes	2.2	1.0-4.6	0.036	1.8	0.8-4	0.12
	No						
Seizure types	1						
	≥ 2	2.03	0.91–4.51	0.77			

## Discussion

This study aimed to determine the proportion of DRE and its associated factors. In this study, we observed a notable prevalence of DRE (14.8%) with almost one-quarter being diagnosed with an epilepsy syndrome. More than three quarter had abnormal neuroimaging findings with cystic encephalomalacia being the most common finding. The onset of seizures during the neonatal period and high initial seizure frequency was found to be independently associated with DRE.

The prevalence of DRE in this cohort was found to be comparable, albeit slightly less, to that previously reported among Nigerian children and adolescents aged 18 years and younger (i.e., 19.9% [5]). The observed similarity could be due to shared risk factors of epilepsy such as birth asphyxia-related complications and CNS infections (e.g. meningitis) [15–17]. Lower rates have been observed in longitudinal studies due to the dynamic nature of DRE .One example is the 15-year follow-up Dutch study, which recorded a significantly lower prevalence (8.5%) [18]. Similarly, a longitudinal Finish study (from 1961 to 1992) showed a reduction of around 5% in the magnitude of DRE by the end of the follow-up period [14]. The pooled prevalence of DRE was found to be 30% however significant variability between different studies can be attributed to different definitions of DRE [3]. The main criteria in the definition of DRE are; length of follow-up period used to assess seizure response and the number of failed AEDs. The duration of the follow-up period varied significantly across studies, ranging from six months to two years [18, 19]. In terms of failed number of AEDs, some studies were more strict on the criteria while others required failure of only one AED [19]. Our study used the current ILAE proposed definition of DRE making our findings reliable for comparison with other centers.

The findings from this study indicate that patients with DRE were more likely to have generalized than focal epilepsies, findings that are similar to a study done in India [16]. On the contrary, studies done in other countries such as USA and Scotland, found the opposite to be true among patients with DRE [7, 17]. A quarter of patients with DRE had infantile or childhood-onset epilepsy syndromes, the commonest being LGS followed by West syndrome, similar to what was observed in India and the Netherlands [13, 16]. Due to overall documented poor seizure control with standard ASM, alternative therapies such as the ketogenic diet have been found to have beneficial results in terms of reducing overall seizure burden [18, 19]. Studies conducted from resource-limited settings revealed a relatively low acceptance of the ketogenic diet. Furthermore there is lack of awareness among healthcare professionals hampering sustainability of this intervention [20, 21]. Due to various reasons ketogenic diet services are not currently accessible at this study site.

Neonatal onset seizures were found to be associated with DRE, similar to what has been observed from other centers [9, 22, 23]. Even though early insult to the brain might predispose patients to develop DRE, evidence also suggests that early presentation might be an intrinsic characteristic of DRE [24]. Drug-resistant neonatal onset seizures often indicate underlying metabolic and genetic causes. While we speculate some of the DRE cases to be attributed to metabolic and genetic causes, diagnostic tests are not available at this center to confirm this. These findings underscore the importance of genetic and metabolic testing for selected cases and timely interventions to decrease epilepsy-related morbidity.

In this study, we noted patients who had higher seizure frequency were likely to be refractory to treatment similar to what was observed from other centers [12, 25, 26]. Repeated seizures have been shown to induce structural and function changes in the brain; which ultimately lead to the formation of recurrent excitatory circuits and therefore increased risk of refractoriness especially for those with infantile-onset seizures [27].

In our study, a substantial proportion of abnormal MRI findings were observed among patients with DRE, aligning with results from other [10, 28]. Cystic encephalomalacia, congenital structural malformations, and brain atrophy predominated in the youngest group less than 10 years of age. Unforeseeably, perinatal brain hypoxia, a prevalent cause of cystic encephalomalacia, did not exhibit an association with DRE possibly due to recall bias leading to an underestimation of severe perinatal events. Notably, children above ten years had distinct imaging findings (e.g., focal gliosis and temporal lobe sclerosis) that qualify them as potential candidates for curative epilepsy surgery [29]. While few epilepsy surgeries have been successfully done in Tanzania, this service remains largely inaccessible due to limited capacity to screen for viable surgical candidates and trained human resources to perform the surgeries.

Despite the observed significant association between abnormal EEG and DRE from other studies, this was not found to be the case in this study [26, 30]. The interictal EEG in this study center is recorded only for 20–30 min and thus offers less chance of picking up epileptiform discharges. Also, the interval between the last seizures to the time of doing the EEG might be prolonged given the logistical issues in our study center, hence decreasing the yield. Furthermore, since we relied on the available reports and not the actual tracings, the investigators could not exclude the possibility of inter-observer variability.

This study had several limitations. First, this was a cross-sectional study limited by the ability to establish a causal effect relationship with the predictors of DRE. Additionally, the relapsing-remitting nature of DRE might lead to over or underestimation of the proportion of DRE, especially given the short inclusion period. Unfortunately due to the sample size of patients with DRE, it was not possible to perform further subgroup analysis on patients using alternative therapies or from consanguineous parents. Finally, some of the variables inquired are subjected to recall bias, with the subsequent possibility of underestimation of certain predictors of DRE.

## Conclusion

DRE is common among patients with epilepsies attending clinics in Tanzania. More than three-quarters of patients with DRE had abnormal neuroimaging with cystic encephalomalacia and brain atrophy being the most common findings. Neonatal onset seizures and high initial seizure frequency were found to be independent predictors of DRE. We recommend patients with neonatal onset seizures and higher initial seizure frequency be followed up closely for early diagnosis of DRE.

## Data Availability

The data set used is not publicly available because the participants have not given consent for public availability. However, the data are available from the corresponding author on reasonable request.

## Abbreviations

ASM: Anti Seizures Medication
CT: Computed Tomography
DRE: Drug-Resistant Epilepsy
EEG: Electroencephalogram
ILAE: International League Against Epilepsy
IRB: Institutional Review Board
LMIC: Low and Middle-Income Countries
MNH: Muhimbili National Hospital
MRI: Magnetic Resonance Imaging
MUHAS: Muhimbili University of Health and Allied Sciences
SD: Standard Deviation
SPSS: Statistical Package For Social Sciences
SSA: Sub Saharan Africa
USA: United States of America
